# PDLIM5 links kidney anion exchanger 1 (kAE1) to ILK and is required for membrane targeting of kAE1

**DOI:** 10.1038/srep39701

**Published:** 2017-01-03

**Authors:** Ya Su, Thomas F. Hiemstra, Yahui Yan, Juan Li, Hannah I. Karet, Lawrence Rosen, Pablo Moreno, Fiona E. Karet Frankl

**Affiliations:** 1 Department of Medical Genetics, University of Cambridge, Cambridge Institute for Medical Research, Cambridge, UK; 2 Department of Medicine, University of Cambridge, Cambridge, UK; 3 Department of Haematology, University of Cambridge, Cambridge, UK.

## Abstract

Anion exchanger 1 (AE1) mediates Cl^−^/HCO_3_^−^ exchange in erythrocytes and kidney intercalated cells where it functions to maintain normal bodily acid-base homeostasis. AE1’s C-terminal tail (AE1C) contains multiple potential membrane targeting/retention determinants, including a predicted PDZ binding motif, which are critical for its normal membrane residency. Here we identify PDLIM5 as a direct binding partner for AE1 in human kidney, via PDLIM5’s PDZ domain and the PDZ binding motif in AE1C. Kidney AE1 (kAE1), PDLIM5 and integrin-linked kinase (ILK) form a multiprotein complex in which PDLIM5 provides a bridge between ILK and AE1C. Depletion of PDLIM5 resulted in significant reduction in kAE1 at the cell membrane, whereas over-expression of kAE1 was accompanied by increased PDLIM5 levels, underscoring the functional importance of PDLIM5 for proper kAE1 membrane residency, as a crucial linker between kAE1 and actin cytoskeleton-associated proteins in polarized cells.

Anion exchanger 1 (AE1) is a sodium-independent member of a family of 1:1 chloride-bicarbonate exchangers. In mammals, AE1 is expressed at high levels in erythrocytes (eAE1) and kidney (kAE1). Under the control of separate promoters, both AE1 isoforms are encoded by *SLC4A1*. Human kAE1 thereby lacks the first 65 residues present in eAE1^1^. In kidney, kAE1 is mainly found at the basolateral membrane of type A acid secreting intercalated cells (α-IC) of the collecting duct in the distal nephron, where it is functionally coupled to the cells’ apical proton pumps to maintain normal bodily acid-base homeostasis. Mutations in *SLC4A1* are associated with distal renal tubular acidosis (dRTA)^2^,^3^, a condition characterized by impaired urinary acid secretion, hyperchloremic metabolic acidosis, hypokalemia, growth retardation, nephrocalcinosis and nephrolithiasis^4^. Mutant kAE1 proteins usually exhibit normal or only modestly reduced Cl^−^/HCO_3_^−^ transport activity, but severe trafficking defects. To date, at least two mistargeting phenotypes, intracellular retention and aberrant membrane accumulation, have been observed and are the major pathogenic mechanism^5^,^6^,^7^,^8^.

AE1 is composed of a large cytosolic N-terminal domain, a central transmembrane anion exchange region predicted to span the lipid bilayer 12–14 times, and a short cytosolic C-terminal tail (AE1C). Although multiple binding sites for proteins such as glycolytic enzymes and cytoskeletal linker proteins have been identified in the N-terminal domain of eAE1^9^,^10^,^11^,^12^, none of these appear to interact with the N-terminus of kAE1^13^,^14^, probably because of structural change caused by truncation of the first 65 residues in kAE1^15^. Notably, dominant dRTA mutations are spread over both transmembrane and C-terminal domains, but no such mutations have been identified within the N-terminus.

AE1’s C-terminus contains 36 residues in which three autosomal dominant dRTA-causing mutations (A888L+889X^16^, M909T^8^ and R901X^17^) have been reported. R901X truncates AE1 by 11 amino acids (R^901^DEYDEVAMPV^911^) (kAE1-Δ11) and has been intensively investigated. In *Xenopus* oocytes, kAE1-Δ11 exhibits normal anion transport function, but is mis-targeted in both Madin-Darby Canine Kidney (MDCK) and HEK293 cells^5^,^6^,^18^, supporting an initial idea that defective urinary acidification arises from trafficking defects of mutant proteins in kidney α-ICs^19^,^20^. The C-terminal region is actually rich in potential targeting motifs/determinants^21^ including a PDZ (**P**ost-synaptic density protein, PSD-95; **D**rosophila disc large tumour suppressor, Dlg; **Z**onula occludens-1, ZO-1) binding motif formed by the last 4 residues (A^908^MPV^911^) that has not been previously characterized. The M909T mutation or deletion of the motif results in trafficking defects of the mutant proteins in polarized kidney epithelial cells, implicating this motif in proper membrane residency of kAE1^8^.

Several proteins have so far been reported to associate with AE1C, and disruption of these interactions results in abnormal cellular location of kAE1^21^,^22^,^23^. We report here a novel C-terminal binding partner, PDLIM5, also called Enigma homolog protein (ENH) and belonging to the Enigma subfamily of PDZ-LIM proteins. PDLIM5 contains one N-terminal PDZ and three C-terminal LIM domains. PDZ domains are a type of common structural domain of 80–90 amino acids and are known as organizers of protein complex assembly. Studies of PDZ proteins in various organisms have provided evidence of their involvement in cellular sorting and targeting (including basolateral membrane targeting) of their binding partners^24^.

## Results

### PDLIM5 is a potential binding partner for kAE1

**Figure 1:**
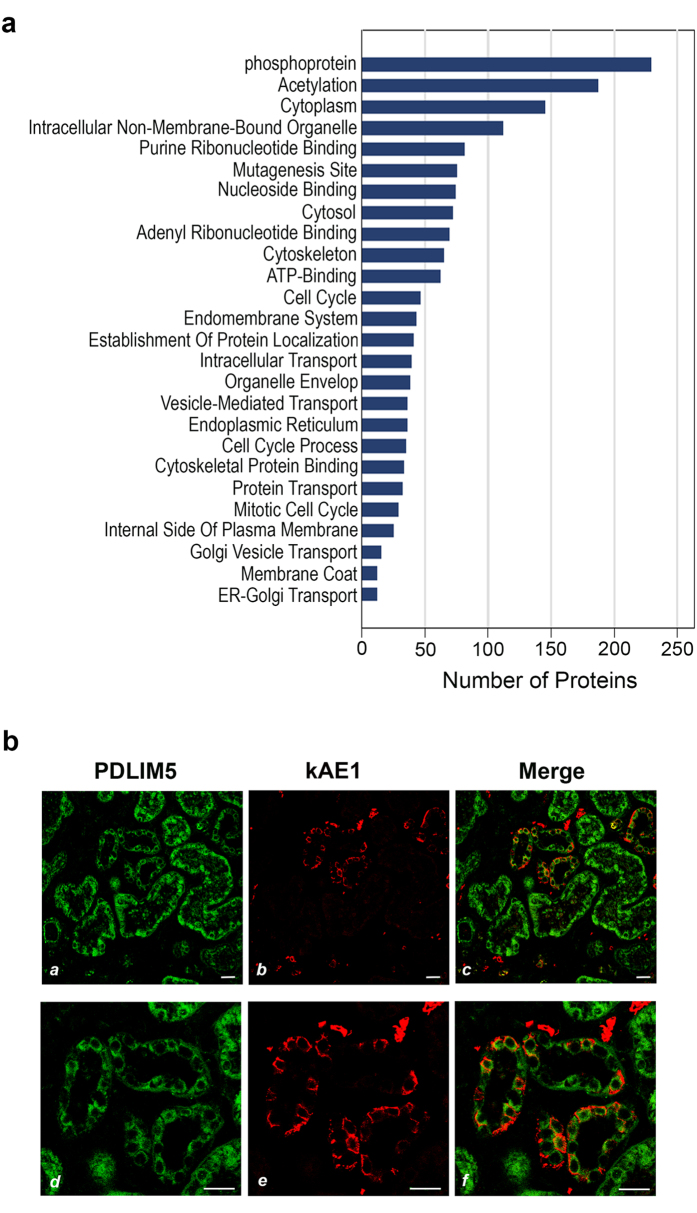
Identification of the PDLIM5 as a potential binding partner for kAE1. (**a**) 480 proteins including PDLIM5, classified into 26 functional enrichment categories, were identified following co-IP-coupled mass spectroscopy from kAE1-expressing cells. (**b**) Immunostaining of normal human kidney cortex (lower panels at high power) showed kAE1 (red, *panels b and e*) basolaterally in intercalated cells. PDLIM5 (green, *panels a and d*) distribution was more widespread throughout nephron segments with some enrichment basolaterally co-localizing with kAE1 (yellow, *panels c and f*). Bars indicate 20 μm.

Co-immunoprecipitation (IP) assays were performed using lysates from MDCK-ΔpMEP-GFP-kAE1 cells with anti-AE1 (Bric-170) as immunoprecipitating antibody and anti-CD63, which does not recognize dog CD63, as a negative control antibody. Mass Spectrometry identified 480 proteins which were classified into 26 functional enrichment categories (Fig. 1a, Supplementary Table 1). Among these potential binding candidates, PDZ domain-containing PDLIM5 was chosen for further investigation.

### PDLIM5 expression in kidney

Lee *et al*. have recently reported gene expression patterns in each of 14 renal tubule segments in rat kidney using RNA-sequencing coupled with classic tubule microdissection^25^. There, PDLIM5 transcripts were found in almost every segment with enhancements in the medullary long descending limb of Henle’s loop, connecting tubule, cortical collecting duct (CD) and inner medullary collecting duct. Dual-immunostaining for kAE1 and PDLIM5 in available sections of human kidney cortex demonstrated known kAE1 localization to the basolateral surface of α-IC in CD (Fig. 1b*, panels b* and *e*) while PDLIM5 was more extensively distributed, including proximal tubules and CD (*panels a* and *d*). Merged confocal optical sections suggested some co-localization of the two proteins in α-IC in the cortical collecting duct (*panels c* and *f)*.

**Figure 2:**
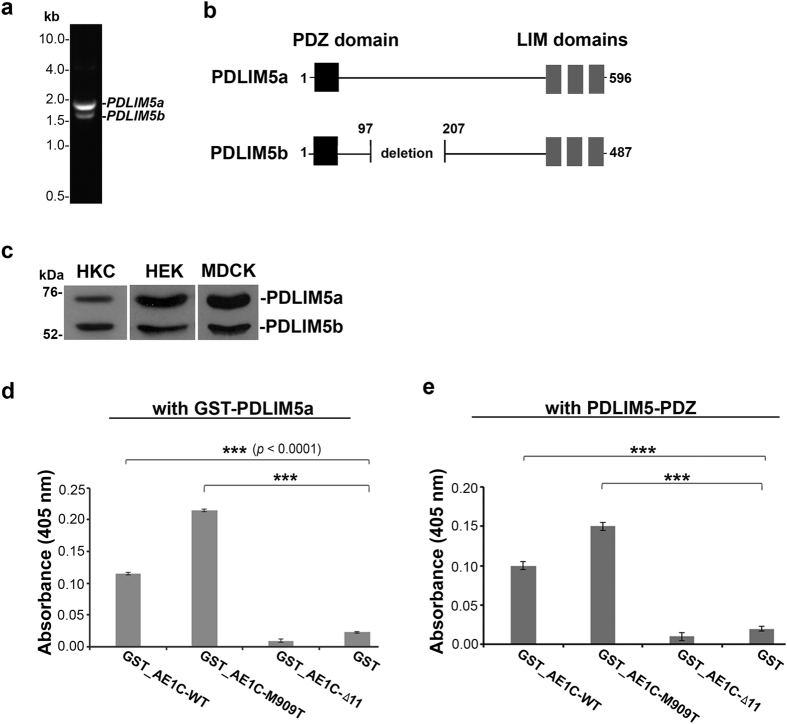
Identification of two major PDLIM5 isoforms in mammalian kidney and *in vitro* confirmation of direct interaction between PDZ domain of PDLIM5 and PDZ binding motif of AE1. (**a**) PCR amplification of human kidney cDNA performed using primers for PDLIM5 coding region. Two fragments, approximately 1.8 and 1.4 kb indicated two *PDLIM5* transcripts that correspond to *PDLIM5a* and *PDLIM5b* respectively. (**b**) Domain organization of PDLIM5a and PDLIM5b proteins. Both isoforms contain one PDZ domain (black) and three LIM domains (grey) at N and C-terminus, respectively, but with a deletion of 109 residues in the middle of PDLIM5b. (**c**) Western blot analysis using anti-PDLIM5-ct detected both PDLIM5a and PDLIM5b isoforms in human kidney cytosol (HKC), HEK-ΔpMEP-eGFP-kAE1 (HEK) and MDCK-ΔpMEP-eGFP-kAE1 (MDCK) cell lysates. (**d**,**e**) ELISA plate coated with GST wild-type (GST_AE1C-WT) or mutant (GST_AE1C-M909T and GST_AE1C-Δ11) fusion protein or GST alone incubated with either GST_PDLIM5 (**d**) or tag-free PDZ domain of PDLIM5 (**e**) followed by detection with anti-PDLIM5-nt antibody. Signals were expressed relative to WT (100%) ± SEM. Specific binding of AE1C-WT or AE1C-M909T to PDLIM5 or PDLIM5-PDZ is shown, demonstrating the importance of the PDZ binding motif in AE1 binding to PDLIM5’s PDZ domain. Signals from GST alone were significantly low (*P* < 0.0001 analyzed using ANOVA) confirming specificity.

PCR products amplified from human kidney cDNA using specific primers displayed two clear bands of 1800 and 1400 bp (Fig. 2a) whose sequences correspond to isoforms a (ID: NP_006448.4) and b (ID: NP_001011513.3) in the NCBI database, respectively. PDLIM5a and PDLIM5b are splicing isoforms; both contain one N-terminal PDZ domain and three C-terminal LIM domains, but differ in the centre where PDLIM5b has 109 residues missing (*panel b*). In addition, both isoforms were identified in all kidney tissue/cell lysates examined (*panel c*) by Western blotting.

### The PDZ binding motif in AE1C is important for direct PDLIM5 binding

To investigate direct protein interactions, we made GST-tagged AE1C-WT, AE1C-Δ11 and AE1C-M909T proteins (Supplementary Figure 1a) as previously described^23^. Expression of tag-free full-length PDLIM5 caused degradation apart from a stable fragment of approximately 13 kD (Supplementary Figure 1b), which N-terminal sequencing showed to be the first 127 residues of PDLIM5 (designated PDLIM5-PDZ), which contains the entire PDZ domain of this protein. We were therefore able to use this domain for ELISA analysis. In addition, we attempted to express and purify a fragment containing the LIM domains of PDLIM5 but this also degraded rapidly during tag cleavage and purification steps.

Parallel ELISA analyses were then performed using AE1C-WT, AE1C-Δ11 or AE1C-M909T GST fusion proteins incubated with GST-PDLIM5a or PDLIM5-PDZ recombinant proteins. AE1C-WT and AE1C-M909T, but not AE1C-Δ11, were able to bind to both tagged PDLIM5a (Fig. 2, *panel d*) and untagged PDLIM5-PDZ (*panel e*). This indicates firstly that A^908^MPV and A^908^TPV at the far C-terminus of AE1 (wild-type and mutant respectively) are both PDZ-binding motifs and secondly that the PDZ domain of PDLIM5 is important for binding.

### PDLIM5 is required for membrane residency of kAE1 in kidney cells

**Figure 3:**
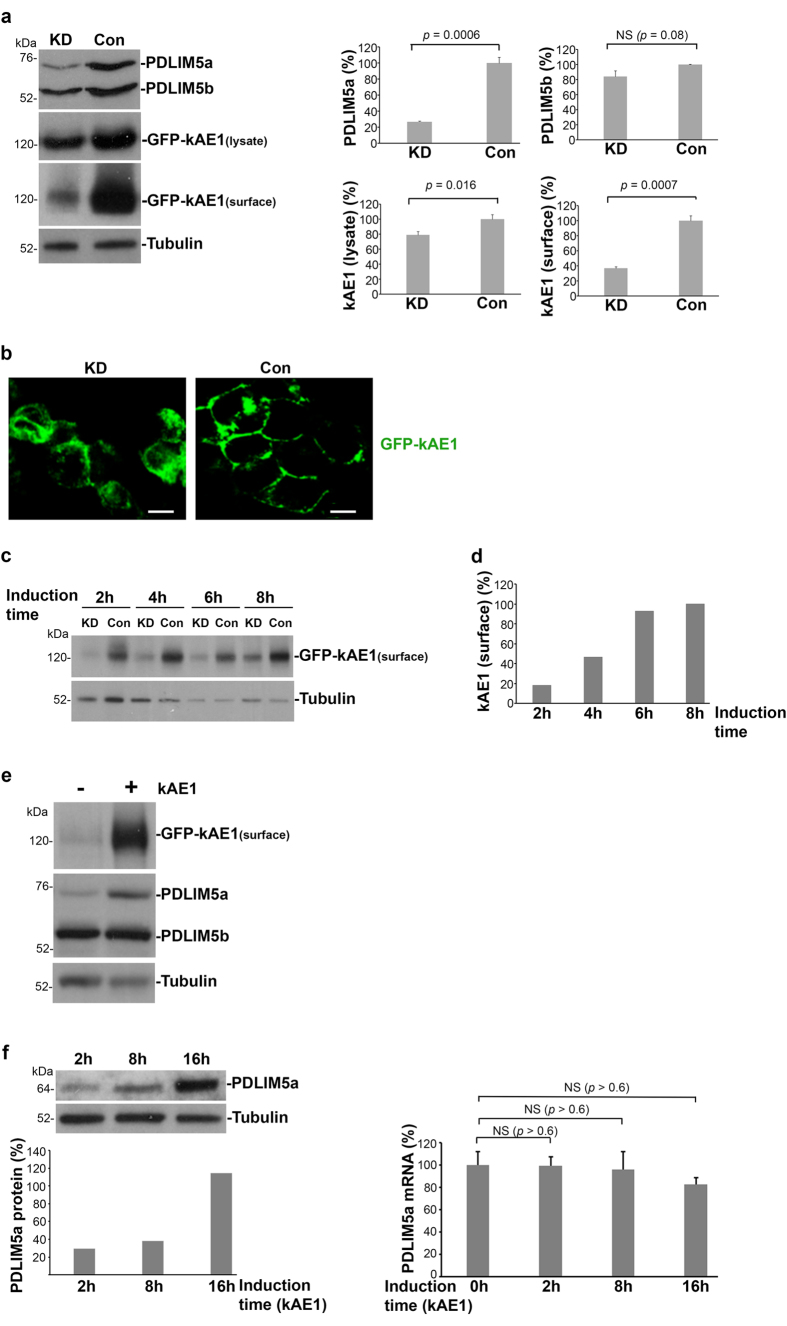
PDLIM5 is required for membrane residency of kAE1. (**a**) Stable HEK-ΔpMEP-eGFP-kAE1 cells were transfected with siRNA against *PDLIM5* (KD) or Control siRNA (Con) over 48 h. Bands generated by Western blot analysis (*left panel*) were densitometrically quantified and expressed relative to WT (100%) ± SEM (*right panels*). Blots are representative of three separate assays. Over 70% knockdown of PDLIM5a protein was achieved. Levels of kAE1 were also severely depleted, especially on the plasma membrane (surface), in knockdown cells. (**b**) Confocal microscopy of stable HEK-ΔpMEP-eGFP-kAE1 cells showed that kAE1 in knockdown cells was significantly intracellular compared to PDLIM5-replete cells. Bars indicate 10 μm. (**c**) Following biotinylation at 2, 4, 6 or 8 hours post-kAE1 induction, membrane levels of kAE1 were severely reduced in knockdown cells at all time-points compared to control cells, where (**d**) kAE1 levels progressively increased. (**e**) kAE1 expression induced in stable HEK-ΔpMEP-eGFP-kAE1 cells (*+lane*) was accompanied by significant increases in levels of PDLIM5 in kAE1-expressing cells compared to kAE1-negative cells (*- lane*). (**f**) Protein and mRNA levels of PDLIM5a/*PDLIM5* were analysed by Western blot (*left panel*) and RTqPCR (*right panel*), respectively, after induction of kAE1 at 2, 8 or 16 hours. Tubulin served as a loading control in panels a, c, e and f.

Since loss of the last 11 residues of AE1 causes not just dRTA/kAE1 mistargeting but also abolishes PDLIM5 binding, we next assessed a possible role for PDLIM5 in kAE1 membrane targeting/retention in kidney cells. We first used siRNA oligonucleotides directed against the 3^rd^ exon of *PDLIM5* (which encodes part of the PDZ domain in both long and short forms) to target endogenous PDLIM5 mRNA in HEK-ΔpMEP-GFP-kAE1 cells. This achieved approximately 70% reduction of endogenous PDLIM5a expression (Fig. 3a), while levels of PDLIM5b were better maintained at about 80%. PDLIM5 depletion resulted in a marked decrease (at least 70%) of kAE1 levels at the cell surface as assessed by biotinylation with accompanying but less marked reduction (~20%) in kAE1 levels in total cell lysates. In concert, confocal microscopy (Fig. 3b) demonstrated a similar reduction of membrane presence in knock down cells, with most kAE1 protein retained intracellularly, suggesting impaired membrane trafficking. As a non-radioactive alternative to pulse-chase analysis, we performed a time-course series at 2, 4, 6 and 8 hours after induction of kAE1 expression in HEK-ΔpMEP-eGFP-kAE1 cells depleted of PDLIM5 (Fig. 3c,d). Surface kAE1 was low in knockdown cells at all time-points, suggesting a defect of membrane targeting rather than achievement of membrane residency followed by internalisation.

A slightly lower level (~50%) of PDLIM5a depletion was achievable in MDCK kAE1-expressing cells, with no reduction in the level of PDLIM5b (Supplementary Figure 2). Interestingly, kAE1 levels were unaffected in the MDCK knock down samples, implying possible compensation by the PDLIM5b isoform for the loss of PDLIM5a in this system.

Second, we examined induction of kAE1 membrane expression in HEK-ΔpMEP-eGFP-kAE1 cells. Steady state was reached at 16 hours (Supplementary Figure 3) and was accompanied by significantly increased total levels of PDLIM5 protein (Fig. 3e), of which the majority was the a-isoform. To differentiate between new PDLIM5 protein synthesis or its stabilization, we examined additional earlier timepoints by real-time quantitative PCR (RTqPCR) and Western blot (Fig. 3f). We found no significant change in mRNA levels for *PDLIM5a (right panel*), whereas PDLIM5 protein levels rose sequentially, suggesting that PDLIM5 is required to stabilise kAE1’s membrane residency.

### PDLIM5, kAE1 and ILK are found in the same complex in human kidney

**Figure 4:**
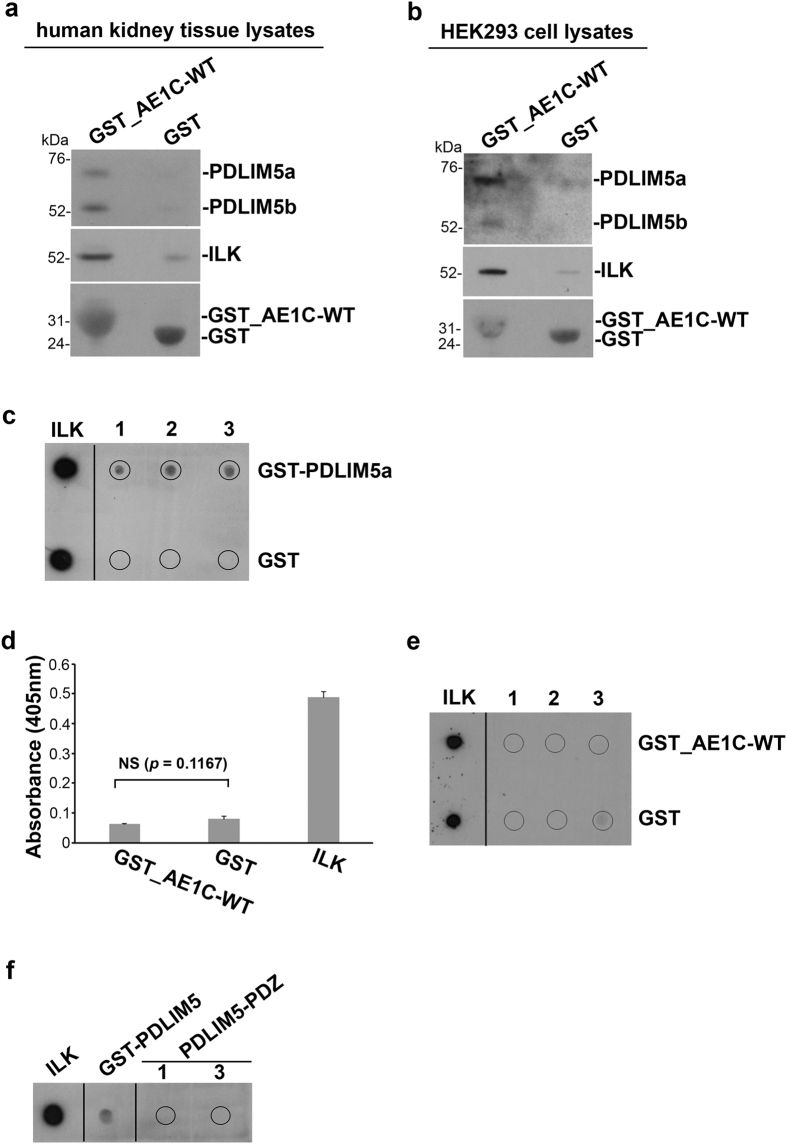
PDLIM5, AE1 and ILK are found in the same complex in human kidney. GST-tagged AE1C-WT or GST alone were employed in bead-bound pull-down assays against human kidney cytosol (**a**) or HEK-ΔpMEP-eGFP-kAE1 cell lysates (**b**). Bound proteins were analyzed by Western blotting using anti-PDLIM5-ct or anti-ILK antibodies, with GST blotting providing loading controls. PDLIM5a, PDLIM5b and ILK were all detected in the precipitates from tissue and cell lysate samples. (**c**) Blot overlay analysis detected ILK binding to PDLIM5 but not to GST using anti-ILK antibody. ILK, 5 pmoles of GST-ILK spotted; 5, 7 and 13 pmoles of GST-PDLIM5 (upper) or GST (lower) were spotted in lanes 1, 2 and 3 respectively. (**d**) ELISA showing absence of ILK binding to either GST_AE1C-WT or GST alone; ILK alone provided a positive control for the anti-ILK antibody (*right bar*). (**e**,**f**) Direct blot overlay analysis confirmed absence of ILK binding to either AE1 or the PDZ domain of PDLIM5 (PDLIM5-PDZ) using anti-ILK antibody. ILK, 2 pmoles of ILK spotted; GST-PDLIM5, 120 pmoles of GST-PDLIM5 spotted (*panel f*); 30, 60, and 120 pmoles of GST-AE1C-WT (*panel e*, upper) or GST (*panel e*, lower) or PDLIM5-PDZ (*panel f*) were spotted in lanes 1, 2 and 3 respectively.

Pull downs using immobilized GST_AE1C-WT fusion protein and incubation with human kidney tissue lysates yielded both a and b isoforms of PDLIM5 (Fig. 4a, representative of three replicate experiments), confirming association of PDLIM5 with the AE1C domain. As integrin linked kinase (ILK) is reported to associate with kAE1 in HEK293 cells and also glomeruli (where a low level of kAE1 has been reported)^26^,^27^, we re-probed the same blot for ILK, with positive results (Fig. 4a). This suggests a possible multiprotein complex formed of kAE1, through its C-terminal domain, with PDLIM5 and ILK.

Similar results were obtained using HEK-ΔpMEP-GFP-kAE1 cell lysates (*panel b*). Finally, a blot overlay assay showed a direct interaction between PDLIM5 and ILK (*panel c*), but neither ELISA (*panel d*) nor blot overlay assays (*panels e/f*) yielded detectable ILK binding to AE1C or the isolated PDZ domain of PDLIM5. Together these data indicate that PDLIM5 forms a bridge between kAE1 and ILK. These results are further supported by bioinformatic analyses (as described in *Methods*) that suggest that the LIM domains in PDLIM5 are capable of interaction with the ankyrin repeats in ILK.

## Discussion

We have here identified PDLIM5 as a binding partner of the terminal 11 residues of kAE1 and demonstrated a direct interaction through PDLIM5’s PDZ domain. Our results suggest that the PDLIM5/kAE1 interaction plays a role in kAE1’s basolateral membrane targeting/retention. The role of the last 11 residues of AE1 for PDLIM5 binding is of particular interest, the presence of basolateral targeting motifs/determinants within this region of AE1 having previously been identified through expression studies of the truncating mutant^5^,^6^,^18^,^28^. In those studies, the mutation led to intracellular retention in HEK293 cells and mis-targeting apically in fully polarized MDCK cells.

We have previously also demonstrated the importance of the far C-terminus for AE1’s membrane residency, firstly by removing just the last 4 residues resulting in intracellular retention, and secondly showing non-polarized targeting with some intracellular retention of kAE1-M909T, a mutation causing dRTA^8^. In that study, we introduced the idea that M909T creates a Type I PDZ binding motif (X(S/T)XΦ, where X and Φ are ‘any’ and ‘hydrophobic’ amino acids, respectively) into the C-terminus. The last four residues (A^908^MPV) in wild-type AE1 are likely a Class II (XΦXΦ) motif^24^. Thus, the preservation of PDLIM5 binding by AE1-M909T or AE1-WT suggests that the PDZ domain in PDLIM5 can function as either Class I or II.

SiRNA-induced depletion of endogenous PDLIM5 led to overall reduction of kAE1 with a major fall in kAE1 levels on the plasma membrane and increased intracellular retention. Furthermore, in the *PDLIM5*-depleted time-course assay, surface kAE1 was low in knockdown cells at all time-points. The overall reduction is likely the result of degradation of non-delivered kAE1 by a lysosomal pathway as described by Almomani *et al*.^29^. Conversely, when membrane overexpression of kAE1 was induced in these cells, significantly increased levels of endogenous PDLIM5 protein, but not mRNA, were observed, suggesting increased protein stability due to PDLIM5’s association with kAE1. Since plasma membrane expression of eGFP-kAE1 reaches steady state at around 14–16 hours after induction of its expression and PDLIM5 levels rise in parallel, we believe that PDLIM5 is involved in both kAE1 translocation to the membrane and in its retention. These results together indicate a requirement of PDLIM5 for the proper membrane residency of kAE1.

The nature of kAE1’s linkage to the underlying actin cytoskeleton has been uncertain. In 2007, Keskanokwong *et al*. reported an association between the kAE1’s N-terminus and ILK via a yeast two-hybrid screen^26^. Further characterization employed co-IPs using various fragments of the two proteins in HEK293 cells. Overexpression of ILK increased kAE1’s presence at the membrane, with a parallel increase in ion transport; pulse-chase assay showed that the two proteins associated early in biosynthesis and travelled together from endoplasmic reticulum to plasma membrane. It was therefore proposed that ILK acts as a bridging molecule between the N-terminus of kAE1 and actin, via paxillin and actopaxin. However, a year later Williamson *et al*. showed that deletion of the majority of kAE1’s proposed ILK binding region failed to alter membrane residency of kAE1^30^, weakening the hypothesis and calling the proposed direct interaction into question. Our GST pulldown results using both human kidney and HEK-ΔpMEP-GFP-kAE1 lysates support Keskanowong’s findings but importantly, implicate the C-terminus of AE1 and not the N-terminus. Our ELISA and/or blot overlay assays, which showed no detectable direct interaction between AE1C and ILK, but a clear interaction between PDLIM5 and ILK that link AE1’s C-terminus to ILK, would account for Williamson’s results.

**Figure 5:**
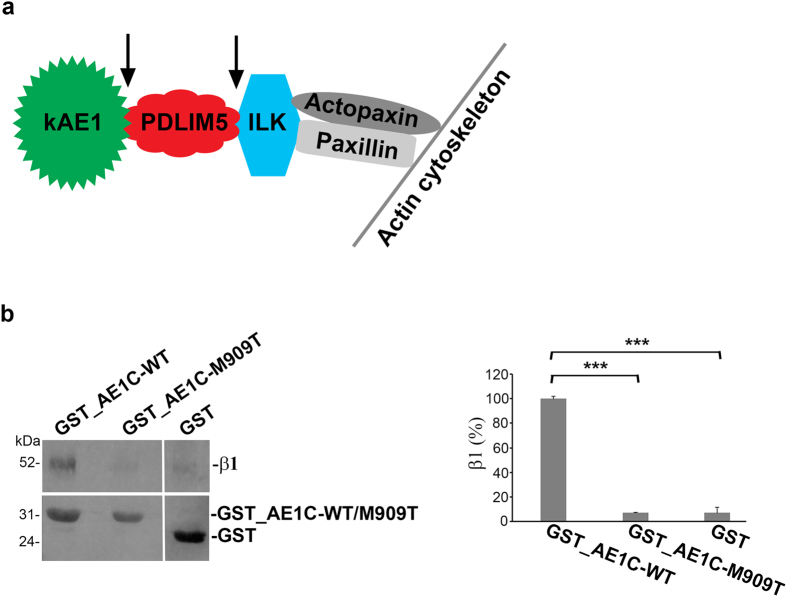
Suggested model for membrane targeting of kAE1, association of the Na^+^, K^+^-ATPase and kAE1 was abolished by AE1-M909T mutation. (**a**) A suggested model for membrane residency of kAE1 based on data from this study (green → red → blue) and reported in^26^,^31^,^32^ (blue → grey). Arrows represent direct interaction beteen the neighboring proteins as reported here. (**b**) GST-tagged AE1C-WT, AE1C-M909T or GST alone were employed in bead-bound pull-down assays against human kidney membrane fractions followed by Western blot analysis to detect the β1 subunit of Na^+^, K^+^-ATPase (*left panel*). Bands were densitometrically quantified and expressed relative to WT (100%) ± SEM, demonstrating disruption of β1 binding by the M909T mutation (*right panel*). Blots are representative of three separate assays.

In thinking about kAE1’s tethering to the actin cytoskeleton, our bioinformatic analysis supports earlier studies reporting that ILK interacts with actopaxin and paxillin^26^,^31^,^32^. Therefore, our working model (Fig. 5a) is one in which PDLIM5 physically tethers kAE1 to ILK, which would be required for correct movement of kAE1 towards its final basolateral membrane destination. Our previous studies of wild-type kAE1 in kidney epithelial cells also demonstrated that it is stabilized on the membrane by Na^+^, K^+^-ATPase through interaction of kAE1 with the pump’s β1 subunit^21^. Our current GST pulldown data (Fig. 5b) indicate that levels of β1 in the AE1C-M909T mutant sample were significantly lower (*P* < 0.0001 by ANOVA) than for wild-type, indicating loss of sodium pump binding by AE1-M909T. This difference may explain why, despite preservation of the PDLIM5 interaction in this mutant and therefore basolaterally directed travel, less appears basolaterally and more is intracellularly retained^8^.

Finally, a number of other basolateral membrane determinants involving residues D^902^EYDEV^907^ are reported within the last 11 residues of AE1^23^,^29^,^30^, most recently including adaptor protein subunit 1B^33^, but how all these determinants collaborate to regulate kAE1 membrane residency is not yet clear. In summary, we have identified a direct interaction between kAE1 and PDLIM5, and our data indicate that PDLIM5 is not only a novel chaperone for kAE1, but also provides a bridge between kAE1 and the underlying actin cytoskeleton. In addition, combining our data with previous reports, a molecular model is emerging of kAE1’s polarized cellular behaviour.

## Methods

### Antisera

The following antibodies were used in this study: Bric-170 (mouse monoclonal, IBGRL 9540, recognizing AE1); anti-PDLIM5-nt (rabbit polyclonal, Abcam ab83060), anti-PDLIM5-mid (rabbit polyclonal, Sigma HPA016740) and anti-PDLIM5-ct (rabbit monoclonal, Bethyl Laboratories A301-704A) – all PDLIM5 specific; anti-integrin linked kinase (ILK) (rabbit monoclonal, Abcam ab76468); anti-CD63 (mouse monoclonal, Abcam ab8219); anti-β1 (mouse monoclonal, recognizing β1 subunit of Na^+^, K^+^-ATPase, Sigma-Aldrich A278) and anti-GST (goat polyclonal, GE healthcare 27-4577-01).

Species-specific horseradish peroxidase (HRP)-conjugated (Dako) and Alexa Fluor^®^ 488 or 568 (Molecular Probes) secondary antibodies were used in Western blot and immunochemistry, respectively.

### Expression constructs

To express AE1 C-terminal domain in *E. coli*, cDNA encoding the last 36 residues of AE1 (AE1C or AE1C-WT) (-L^876^IFRNVELQCLDADDAKATFDEEEGRDEYDEVAMPV^911^), or truncating the last 11 residues of AE1C (AE1C-Δ11), or AE1C with a missense mutation M909T (AE1C-M909T) were each cloned into pGEX-4T-1 vector to create N-terminal GST-tagged AE1C-WT, AE1C-Δ11 or AE1C-M909T constructs as previously described^8^,^23^.

cDNA encoding PDLIM5 was amplified from human kidney cDNA pool by high fidelity PCR using primers PDLIM5_F CCGGAGCTCATGAGCAACTACAGTGTGTCA and PDLIM5_R CCGGCTCGA-GTCAAAAATTCACAGAATGAGCATG to introduce *Sac*I and *Xho*I restriction sites, respectively. These sites were used to clone the PCR product into pSUMO3 vector (LifeSensors, Inc.) which contains His_6_-SUMO3 double tags upstream of a *Sac*I site. All constructs were sequence-verified prior to use. To express intact kAE1 in mammalian cells, full-length cDNA was cloned into inducible vector ΔpMEP4 to create N-terminal eGFP-tagged kAE1 (ΔpMEP-eGFP-kAE1) as previously described^8^,^21^.

### Protein expression and purification in *E. coli* cells

GST-tagged AE1C-WT (GST_AE1C-WT), AE1C-Δ11 (GST_AE1C-Δ11) or AE1C-M909T (GST_AE1C-M909T) fusion proteins were expressed in *E. coli* BL21 cells and purified using glutathione sepharose beads^34^.

cDNA encoding PDLIM5 within pSUMO3 vector was expressed in *E. coli* BL21 cells to produce an N-terminal His_6_-SUMO3 tagged PDLIM5 fusion protein, which was purified using Ni-NTA agarose resin (QIAGEN). Eluate containing 200 mM imidazole (pH 7.4) from the Ni-NTA resin was digested with SUMO proteinase 2 (LifeSensors, Inc.) to remove the His_6_-SUMO3 tag. Digests were further purified by size-exclusion chromatography using HiLoad 26/60 Superdex 75 (GE life science) column equilibrated with PBS. Fractions collected were initially analyzed by SDS-PAGE coomassie staining, then Western blotting and N-terminal sequencing (Department of Biochemistry, University of Cambridge).

### Cell culture, cell lysis and cell surface biotinylation

Cells were grown and maintained in DMEM (Sigma-Aldrich) supplemented with FBS (10%), penicillin (100 U/ml)/streptomycin (100 μg/ml) and L-glutamine (2 mM) at 37 °C with 95% air and 5% CO_2_.

Cell lysates were collected in various buffers depending on experiments. For co-IP and GST pull-down assays (MDCK-ΔpMEP-GFP-kAE1 and HEK-ΔpMEP-GFP-kAE1 cells respectively), buffer contained 150 mM NaCl, 20 mM Tris-HCl (pH 7.4), 10% glycerol, 1% Nonidet P40 (NP-40), 2 mM PMSF and EDTA-free Protease Inhibitor Cocktail (Roche) and for Western blot and cell surface biotinylation analyses, 50 mM Tris-HCl (pH 7.4), 150 mM NaCl, 5 mM EDTA, 1% NP-40 and 0.5% sodium deoxycholate with Protease Inhibitor Cocktail.

To examine levels of kAE1 at the plasma membrane, cell surface biotinylation was performed using the Cell Surface Protein Isolation Kit (Pierce) according to manufacturer’s instructions.

### Plasmid or siRNA transfection

MDCKII and HEK293 express endogenous PDLIM5, but not AE1. To overexpress kAE1, both lines were transfected with ΔpMEP-GFP-kAE1 followed by stable clone selection, and maintenance as described^21^.

Endogenous PDLIM5 expression in stable HEK-ΔpMEP-eGFP-kAE1 cells was depleted with a specific siRNA oligonucleotide (ID: 17950, Ambion). This, or a *Silencer*^®^ Negative Control #1 siRNA (Ambion) was transfected at a final concentration of 50 nM using Lipofectamine™ RNAiMAX reagents (Invitrogen) according to manufacturer’s instructions. Analysis was conducted 48 h later.

### Protein expression in mammalian cells

Stable MDCK-ΔpMEP-GFP-kAE1 cells were seeded on Corning^®^ Transwell^®^ polycarbonate membrane cell culture inserts (Transwell filters, Corning Life Sciences) and grown for 4 days to form polarized monolayers. HEK-ΔpMEP-GFP-kAE1 cells were grown either on glass coverslips or in normal tissue culture plates/flasks. GFP-tagged kAE1 protein expression was induced for 8–12 h as previously described^8^,^21^.

### Immunofluorescence microscopy

Ethically-approved and formally patient-consented samples of normal human kidney were obtained from the Addenbrooke’s Hospital Tissue Bank (Cambridge Research Ethics Committee approval 03/279). All studies were carried out in accordance with relevant guidelines and regulations. 4% formaldehyde-fixed paraffin wax-embedded 5 μm-thick kidney sections were dual-immunostained for kAE1 and PDLIM5 based on previously described methods^35^. Following citrate buffer antigen retrieval, sections were blocked with 10% FBS in PBS containing 0.01% Tween 20 (blocking buffer-1), then incubated with primary antibodies (Bric-170 and anti-PDLIM5-mid), at 1:100 dilution at 4 °C overnight. Fluorochrome-conjugated secondary antibodies were used at 1:500 dilution for 1 h at room temperature. Sections were mounted in Vectashield Mounting Medium (Vector Laboratories) and examined with a Confocal Laser Scanning Microscope (LSM880). As controls, primary antibodies were replaced by either non-immune serum or isotype-specific antisera; all steps were followed unchanged.

### Co-IP-coupled Mass Spectrometry and GST pull-down assays

For co-IP assays using MDCK cells stably expressing full-length kAE1^8^,^21^,^23^, cell lysates were pre-cleared and transferred to protein G-agarose beads preloaded with mouse monoclonal antibodies Bric-170 or anti-CD63 (which does not cross-react with dog CD63) for overnight incubation at 4 °C. Beads were then thoroughly washed with co-IP lysis buffer containing reduced NP-40 (0.1%). Proteins co-immunoprecipitated were separated by SDS-PAGE. Proteomic analyses were carried out at the Cambridge Centre for Proteomics. All gel fragments generated were excised and subjected to to Liquid Chromatography tandem Mass Spectrometry (LC-MS/MS) after in-gel trypsin digestion. LC-MS/MS was performed using an Eksigent NanoLC-1D Plus (Eksigent Technologies) HPLC system and an LTQ Orbitrap Mass Spectrometer (Thermo Fisher Scientific), as described in detail elsewhere^36^. Digests from gel segments were run with dynamic exclusion. MS data were processed using the SEQUEST Bioworks Browser (version 3.3.1 SP1; Thermo Fisher Scientific) to generate MS/MS peak lists. Combined peak list files were submitted to the MASCOT search algorithm (version 2.2.1; Matrix Science) and searched against the IPI-Human Database, version 4.3. All ambiguous peptides were excluded unless matched only to products of a single gene. Protein identification required at least two unique peptides, with a false discovery rate of <0.1. In addition, the DAVID bioinformatics tool was used to generate functional enrichment categories with a false discovery rate of <0.01.

GST-pull down assays using cytosol or membrane fractions of human kidney cortex lysates prepared as described^37^, or lysates from HEK-ΔpMEP-GFP-kAE1 cells were carried out as described^38^. Bound proteins were probed on blots with anti-PDLIM5-ct and anti-ILK antibodies, and band intensity measured using ImageJ software (National Institutes of Health). The same blot was re-probed with anti-β1, instead of anti-GST antibody, as loading control (Supplementary Figure 4).

### ELISA and blot overlay analysis

ELISA was performed^23^ by immobilizing 100 μl of 30 μM of GST_AE1C-WT, GST_AE1C-Δ11, GST_AE1C-M909T or GST alone onto a 96-well plate and incubating with 100 μl of 2 μg/ml of GST_PDLIM5 (AbNova, H00010611-P01) or tag-free PDLIM5-PDZ or GST_ILK (AbNova, H00003611-P01) recombinant protein. Detection was with anti-PDLIM5-nt or anti-ILK and HRP conjugated antibodies, and signals were visualized with ABTS.

Blot overlay assay was carried out^39^ using equal moles (at pmole level, see Fig. 4 legend for details) of one of GST-AE1C, GST-PDLIM5, PDLIM5-PDZ or GST alone spotted onto gridded 0.45 μm Nitrocellulose Transfer Membrane (Whatman International Ltd) and air dried for 3 h. Following blocking in PBS containing 2% skimmed milk and 0.1% Tween 20 (blocking buffer-2), the membrane was first overlaid with GST-ILK recombinant protein (50–65 pmoles), then washed and detected with anti-ILK and HRP conjugated antibodies. Blocking buffer-2 was used for all washes and for dilutions of the ligand and antibodies.

### Real-time quantitative PCR

HEK-ΔpMEP-GFP-kAE1 cells were collected at 0, 2, 8 and 16 hours after GFP-tagged kAE1 protein expression was induced. Total RNA was extracted using Tri Reagent (Sigma-Aldrich) and treated with DNase I (Invitrogen), and first strand cDNA was synthesised using superscript III (Invitrogen) according to the manufacturer’s instructions. RTqPCR was performed for *PDLIM5* expression using qPCR SYBR Green (Agilent Technologies UK Ltd.) and Mx3000P^TM^ Real-Time PCR system (Stratagene). Forward and reverse primers for human *PDLIM5* were 5′-CCGGTTCCTGTTCAAAAGGG-3′ and 5′-GCCGTGGTGCCTTATTGTAG-3′, respectively. *PDLIM5* expression levels were calculated using the delta-delta-Ct method relative to β-Actin (*ACTB*) expression. Forward and reverse primers for human *ACTB* were 5′-CCCTGGAGAAGAGCTACGAG-3′ and 5′- AGGTAGTTTCGTGGATGCCA-3′, respectively.

### Statistical analyses

Statistical analyses were performed using either unpaired Student’s *T* tests or ANOVA.

### Bioinformatic analysis

String-DB [ http://www.ncbi.nlm.nih.gov/pubmed/25352553] was searched using Homo sapiens gene names for PDLIM5, ILK, paxillin and actopaxin. DOMINE^40^ and InterPro databases were used to seek evidence of interaction between the domains of every possible pair of proteins among them, domains of each protein having been collected from the Interpro^41^ database.

## Additional Information

**How to cite this article:** Su, Y. *et al*. PDLIM5 links kidney anion exchanger 1 (kAE1) to ILK and is required for membrane targeting of kAE1. *Sci. Rep.* **7**, 39701; doi: 10.1038/srep39701 (2017).

**Publisher's note:** Springer Nature remains neutral with regard to jurisdictional claims in published maps and institutional affiliations.

## Supplementary Material

Supplementary Figures

Supplementary Dataset 1
