# Supplementary material for: PDLIM5 links kidney anion exchanger 1 (kAE1) to ILK and is required for membrane targeting of kAE1
Source: Sci Rep. 2017 Jan 3;7:39701. doi: 10.1038/srep39701 (PMC5206653; doi:10.1038/srep39701)
Supplement: Supplementary Figures [file srep39701-s1.pdf]

PDLIM5 links kidney anion exchanger 1 (kAE1) to ILK and is required for membrane targeting of kAE1.

SUPPLEMENTARY FIGURES

Ya Su[1], Thomas F Hiemstra[2], Yahui Yan[3], Juan Li[3], Hannah I Karet[1], Lawrence Rosen[1], Pablo Moreno[1] and Fiona E Karet Frankl[1]

Departments of [1]Medical Genetics, [2] Medicine, [3] Hematology, University of Cambridge.

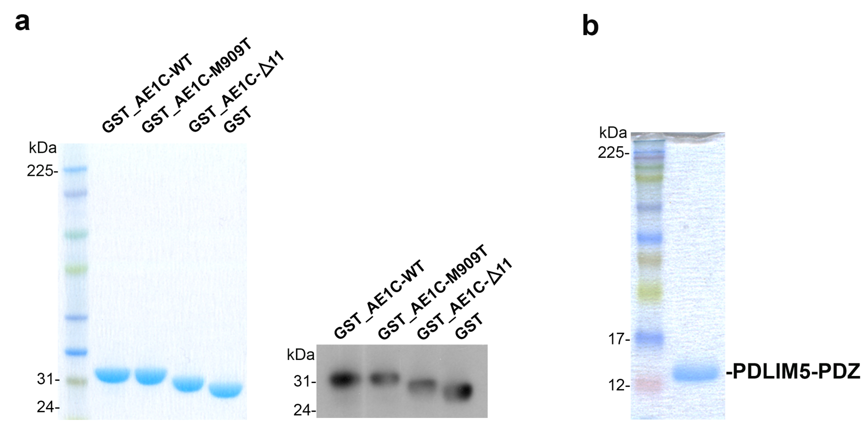

Su *et al.* Supplementary Figure 1

(**a**) Coomassie stain and Western blotting of:
GST fusion proteins (GST_AE1C-WT, GST_AE1C-M909T and GST_AE1C- Δ11) or GST alone used in ELISA, blot overlay and pull down assays.
(b) Coomassie stain of gel filtration-purified and N-terminal sequencing confirmed tag-free PDZ domian of PDLIM5 (PDLIM5-PDZ).

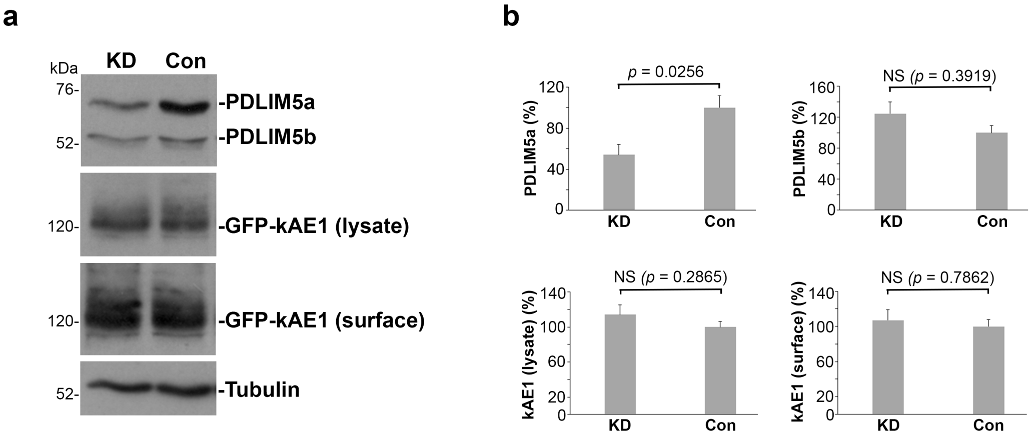

Su *et al.* Supplementary Figure 2

MDCK-ΔpMEP-eGFP-kAE1 cells were transfected with siRNA against *PDLIM5* (KD) or a nonspecific siRNA (Silencer® Negative Control #4 siRNA, Ambion) (Con) using the Cell Line Nucleofection Kit L (Amaxa) over 2-3 days. Plasma membrane proteins (surface) and total cell lysates (lysate) were prepared and analyzed by Western blotting (**a**) as described in Methods. (**b**) Densitometric analysis of bands in (a) were expressed relative to WT (100%) ± SEM. Detection with anti-PDLIM5-ct antibody showed approximately 50% knockdown of PDLIM5a protein was achieved, but no reduction in the level of PDLIM5b protein was observed in knock-down cells. Detection with Bric170 (anti-AE1) antibody showed preservation of kAE1 levels in both total cell lysates and cell surface fractions in the knock-down cells, suggesting that PDLIM5b might be able to compensate for the reduction of PDLIM5a.

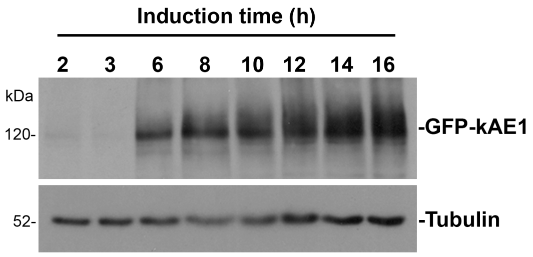

Su *et al*. Supplementary Figure 3

Stable HEK-ΔpMEP-eGFP-kAE1 cells were grown in T-75 flasks under normal conditions. GFP-tagged kAE1 expression was induced for 2, 3, 6, 8, 10, 12, 14, or 16 h. Cell surface proteins were isolated by biotinylation, then separated by SDS-PAGE followed by Western blot analysis of the same blot using Bric-170 (anti-AE1) or anti-Tubulin antibody as described in ‘Methods’. Steady state surface expression of eGFP-kAE1 was observed (based on densitometric analysis) by 14 h.

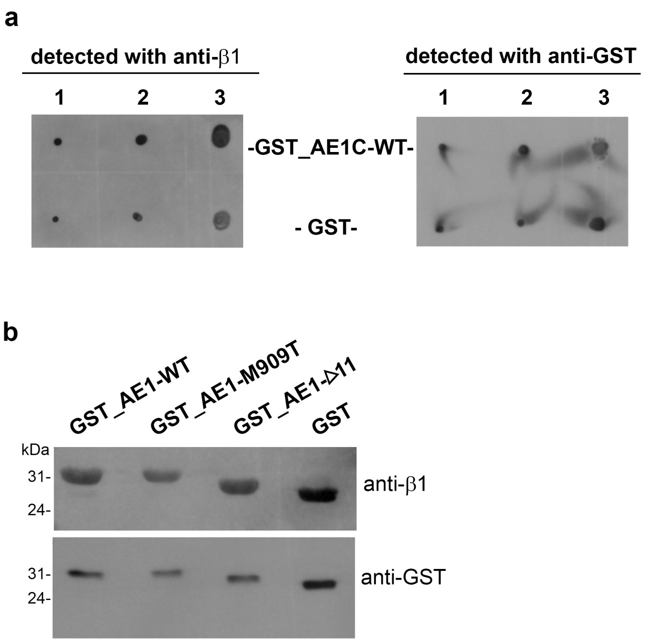

Su *et al.* Supplementary Figure 4

Comparison of sensitivity of cross-reaction of anti-β1 antibody to GST fusion proteins and GST.

In this study, we used anti-β1 instead anti-GST antibody to probe GST in pull-down assays, this is because firstly when reprobing the same blot with anti-GST antibody, band intensity generated was too strong due to relatively higher amount of precipitates necessary to be loaded for detection of the proteins of interest. Secondly, we have noticed that the anti-β1 antibody cross-reacts with GST, and also sensitivities of the cross-reaction of this antibody to GST and GST-AE1C fusion proteins are almost equal.

(**a**) 65 pmole (1), 130 pmole (2), or 325 pmole (3) of each GST_AE1-WT or GST protein was spotted on nitrocellulose membrane followed by detection using anti-β1 (*left panel*) or anti-GST (*right panel*) antibody. The anti-β1 blot was exposed to film for approximately 20 min; the anti-GST blot was exposed to a pile of three films for appoximately 1 sec and the top film is shown.
(**b**) Blot containing co-precipitated protein samples from a GST pull-down assay using GST fusion proteins (GST_AE1C-WT, GST_AE1C-M909T, GST_AE1CΔ11) or GST alone incubated with human kidney tissue lysates was probed with anti-β1 antibody (*upper panel*, bands represent corresponding GST fusion and GST proteins). Each of the pull-down samples was first diluted 1:100, then reloaded and bloted with anti-GST antibody (*lower panel*). Films were exposed to both blots for approximately 1 min.
